# SenseJoy, a pluggable solution for assessing user behavior during powered wheelchair driving tasks

**DOI:** 10.1186/s12984-019-0613-x

**Published:** 2019-11-06

**Authors:** Olivier Rabreau, Sylvain Chevallier, Luc Chassagne, Eric Monacelli

**Affiliations:** LISV laboratory, University of Versailles Saint-Quentin, 10-12 avenue de l’Europe, Vélizy, France

**Keywords:** Powered wheelchair prescription, Joystick driving, Performance assessment, Data logger, Clustering method

## Abstract

**Background:**

The complex task of Electric Powered Wheelchairs (EPW) prescription relies mainly on personal experience and subjective observations despite standardized processes and protocols. The most informative measurements come from joystick monitoring, but recording direct joystick outputs require to disassemble the joystick. We propose a new solution called “SenseJoy” that is easy to plug on a joystick and is suitable to characterize the driver behavior by estimating the joystick command.

**Methods:**

SenseJoy is a pluggable system embedded on EPW built with a 3D accelerometer and a 2D gyrometer placed within the joystick and another 3D accelerometer located at the basis of the joystick. Data is sampled at 39 Hz and processed offline. First, SenseJoy sensitivity is assessed on wheelchair driving tasks performed by a group of 8 drivers (31 ± 8 years old, including one driver with left hemiplegia, one with cerebral palsy) in a lab environment. Direct joystick measurements are compared with SenseJoy estimations in different driving exercises. A second group of 5 drivers is recorded in the ecological context of a rehabilitation center (41 ± 10 years old, with two tetraplegic drivers, one tetraplegic driver with cognitive disorder, one driver post-stroke, one driver with right hemiplegia). The measurements from all groups of drivers are evaluated with an unsupervised statistical analysis, to estimate driving profile clusters.

**Results:**

The SenseJoy is able to measure the EPW joystick inclination angles with a resolution of 1.31% and 1.23% in backward/forward and left/right directions respectively. A statistical validation ensures that the classical joystick-based indicators are equivalent when acquired with the SenseJoy or with a direct joystick output connection. Using an unsupervised methodology, based on a similarity matrix between subjects, it is possible to characterize the driver profile from real data.

**Conclusion:**

SenseJoy is a pluggable system for assessing the joystick controls during EPW driving tasks. This system can be plugged on any EPW equipped with a joystick control interface. We demonstrate that it correctly estimates the performance indicators and it is able to characterize driving profile. The system is suitable and efficient to assist therapists in their recommendation, by providing objective measures with a fast installation process.

## Background

Chronic conditions that impede the mobility have an impact on the quality of life. One can rely on mobility assistive devices to improve the well-being, such as manual wheelchairs, power wheelchairs, scooters or other motorized vehicles [1]. A correct compatibility between the driver and his mobility device is a crucial point to be beneficial and to ensure a noticeable social impact. Studies with wheelchair users indicate that 70% of users had a skill score of less than 30% [2], leaving room for improvement. Many users even had complaints about their wheelchairs and, in the case of Electric Powered Wheelchairs (EPW), it is almost half the population [3].

Therapists play a central role in wheelchair prescription, ensuring the efficient interaction between individuals and their technical aids. The prescription task is a complex and challenging intervention, in order to select the appropriate wheelchair with correct settings. This complexity arises from the relationship between the wheelchair users – characterized by their needs, abilities and preferences – the available technology and the constraints of the considered environment for mobility [4]. Prescriptions that do not meet the users’ expectation often result in neglecting the equipment [5]. After the prescription, therapists have to monitor and to assess the user during her learning phase. Depending on the pathology, the maneuverability and the wheelchair encumbrance, wheelchair driving is often a challenging task for the user [6].

**Fig. 1:**
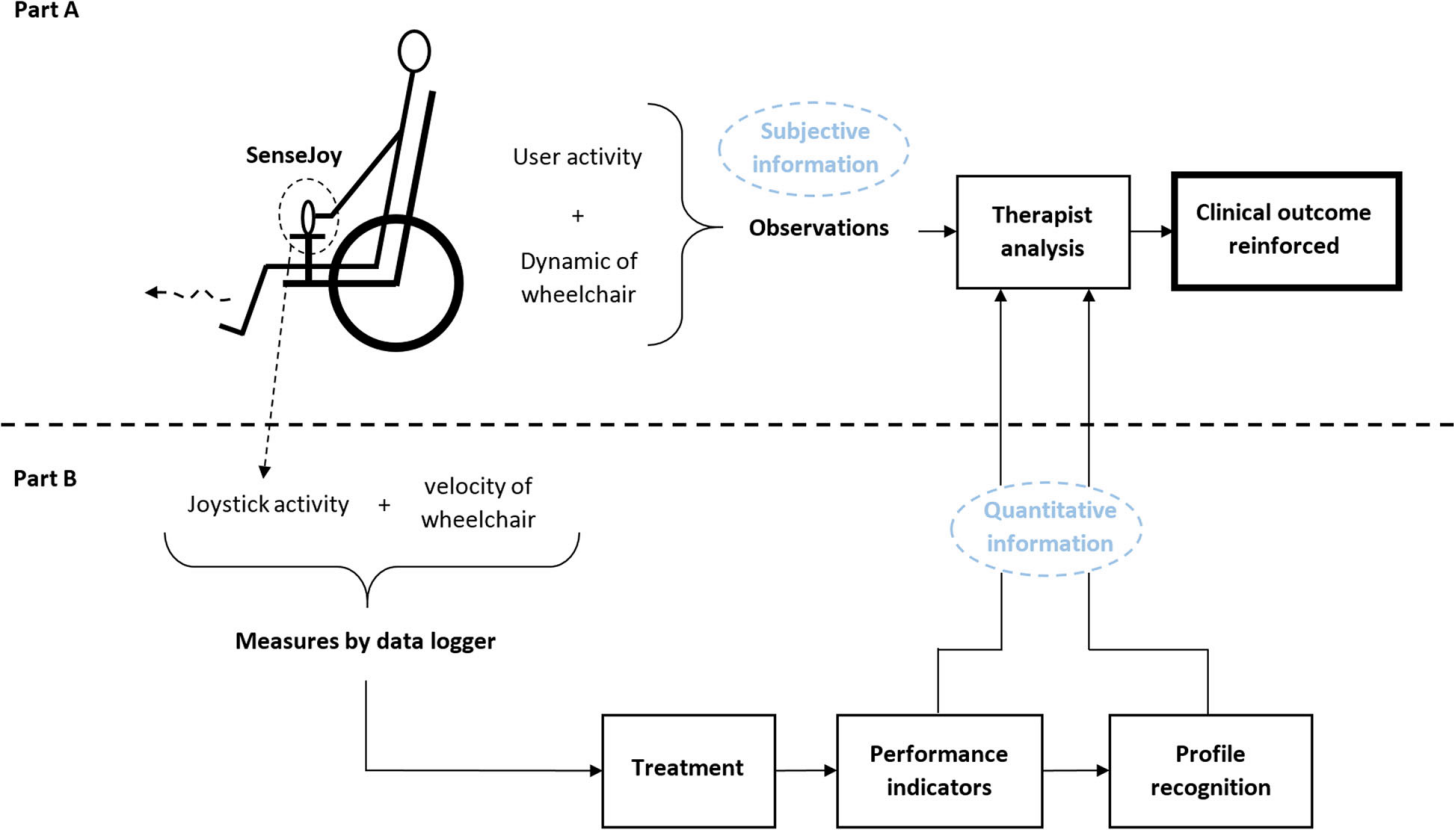
Part A: Standard clinical assessment for a wheelchair prescription. Part B: Reinforced clinical outcomes with data from EPW data loggers

Several solutions exist to assist and improve the technical aid prescription: standardized processes [7], guidelines adoption [8] or protocols such as the Wheelchair Skill Test (WST) [9]. By creating a common methodological framework, these solutions assist therapists as shown in Fig. 1-A. However, personal experience and subjective observations still play a critical role for prescriptions and monitoring.

Wheelchair data loggers are increasingly popular, they provide a quantitative assessment of wheelchair users’ activities [10, 11]. Those innovative technological solutions produce objective measurements that reduce subjective factors in therapists’ analysis [12]. Data loggers are either embedded on the wheelchair or seldom positioned directly on the user, to measure different types of information with accelerometers, pressure sensors, odometers, etc [6, 10, 11, 13, 14]. Most meaningful indicators come from the joystick monitoring on the monitoring of the joystick control [6] to provide a precise impact analysis [13]. Existing joystick monitoring solutions are direct, in the sense that they require to disassemble the joystick to record output values. This solution is not viable as it void the warranty of the user wheelchair and there is a risk of the damaging the EPW.

We propose in this study a novel pluggable solution named *SenseJoy* for monitoring and assessing the joystick controls during driving tasks, as shown on Fig. 1-B. Joystick displacements are a primary source of information to assess the EPW driving abilities of a user. SenseJoy includes a dedicated hardware part, that is easy to plug on the joystick, and monitors the user activity. Unlike existing solutions that require to disassemble the joystick, our system is non-destructive and completely transparent for the EPW users. SenseJoy provides meaningful information to the therapist, in order to produce a reinforced clinical outcome.

Our contributions are twofold:

- An easy-to-plug system, compatible with any joystick-driven EPW
- A novel clustering method to characterize the driver profile, based on the WST results.

These contributions are supported by a metrological validation and an analysis with 13 subjects, including 7 people with disabilities. The ecological part of the evaluation is conducted in the occupational therapy department of Bobigny rehabilitation center (France).

## Method

### Proposed approach

**Fig. 2:**
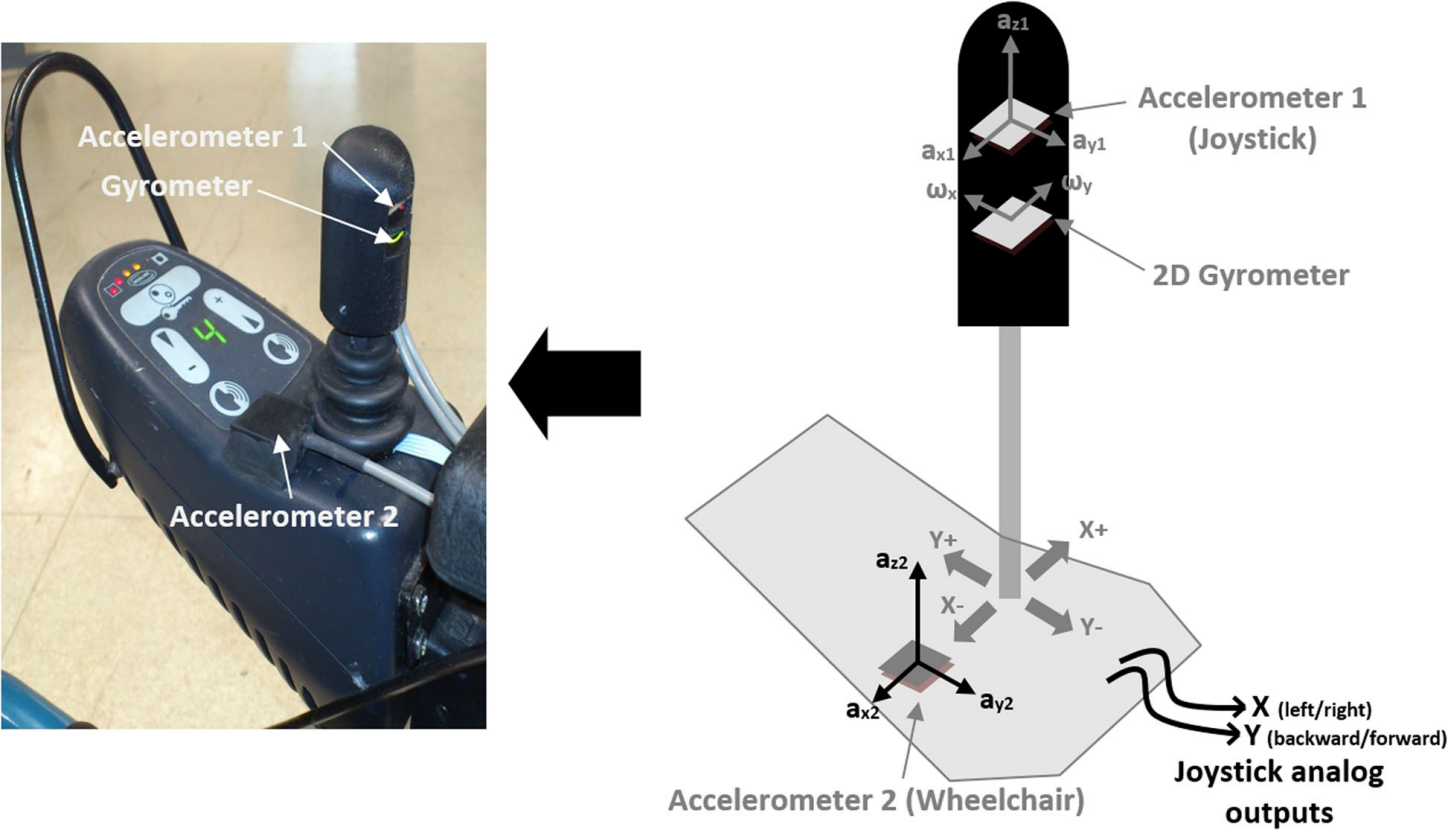
The SenseJoy system mounted on an EPW joystick. The SenseJoy contains a 3D accelerometer and a 2D gyrometer used for measuring joystick movements; another 3D accelerometer is located on the joystick box near the joystick for measuring wheelchair movement

The SenseJoy solution is a stick to mount in place of any joystick grip part, with a remote component placed on the side. The pluggable part is designed to ensure the precise location of sensors and is 3D-printed. The system integrates two 3D analog accelerometers (ADXL335 from National Instrument) and a 2D analog gyrometer (ITG1215 from Invensense). An accelerometer (accelerometer 2 on as shown on Fig. 2) is located on the wheelchair, close to the joystick, and another (accelerometer 1) is located within the joystick. The gyrometer is also located within the joystick, just under the accelerometer 1. The two accelerometers are aligned in same direction and their Y-axes are aligned in the forward-backward axis of the wheelchair. The X-axis is aligned with the left side-right side axis. It is better to ensure a correct alignment of the inertial sensors with the wheelchair movement direction to obtain the highest acceleration range and so to make the most of the sensor sensitivity.

Digital data acquisition is made by a microcontroller card, designed in our lab and based on PIC18F2550 from Microchip. The sampling frequency is 39 Hz. Each sample is composed of eight values : six 3D accelerometer components (*a*_*x*1_, *a*_*y*1_, *a*_*z*1_, *a*_*x*2_, *a*_*y*2_ and *a*_*z*2_) and two angular velocities (*ω*_*x*_, *ω*_*y*_), as indicated on Fig. 2. All data are sent by USB bus to a laptop and saved in a text file by a data logger software (Sniffer) for an offline analysis.

### Data processing

The data are analyzed offline using custom routines developed in Matlab (MathWorks, USA). The first step is to normalize the 3D accelerometer components between -1 and +1. This normalization requires a calibration step to determine the minimum and maximum values measured with the EPW parked on a horizontal ground. In a second step, the difference between accelerometers 1 and 2 minimize the effects of EPW acceleration on the joystick:

1 $$ \vec{A}= \left(\begin{array}{l} a_{x} \\ a_{y} \\ a_{z} \end{array} \right) = \left(\begin{array}{l} a_{x2}-a_{x1} \\ a_{y2}-a_{y1} \\ a_{z2}-a_{z1} \end{array} \right)  $$

**Fig. 3:**
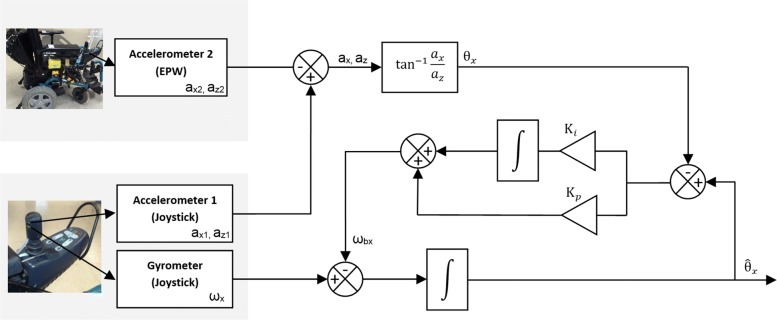
The block diagram of the complementary filtering for the left/right joystick axis. Differential acceleration between joystick and EPW is used to calculate the angular variation in the left/right joystick axis. This value is compared to the estimated angle variation updated by gyrometer measurement and corrected with the previous angle estimation. The same operations are done for the backward/forward axis

This simple processing does not remove completely the effects of the EPW movements in all situations, especially when translation and rotation movements are combined, but reduces them. This simple yet effective processing yields recording with a sufficient precision for an assessment during a WST protocol. The resulting vector could be seen as an approximation of the linear accelerations of user’s actions on the joystick. After applying a one-dimensional median filter (order 20) to remove outlier values, we determine the angular variation for each axis of the bidirectional joystick as follows:

2 $$ \theta_{x}=\arctan \frac{a_{x}}{a_{z}} \text{ and} \theta_{y}=\arctan \frac{a_{y}}{a_{z}}  $$

where *θ*_*x*_ express the angular variation of the joystick in the left/right direction and *θ*_*y*_ in the backward/forward direction. To improve the angular variation estimation, we combine the acceleration data from accelerometers and the angular velocity data from the gyrometer with a complementary filter that is described on Fig. 3. The resulting angles are expressed in Laplace form:

3 $$ {\hat\theta_{x}}=\frac{1}{S}(\omega_{x}-\omega_{b_{x}})=\frac{1}{S}\omega_{x}+ \frac{K_{p}}{S}(\theta_{x}-\hat\theta_{x})+\frac{K_{i}}{S^{2}}(\theta_{x}- \hat\theta_{x})  $$

4 $$ {\hat\theta_{y}}=\frac{1}{S}(\omega_{y}-\omega_{b_{y}})=\frac{1}{S}\omega_{y}+ \frac{K_{p}}{S}(\theta_{y}-\hat\theta_{y})+\frac{K_{i}}{S^{2}}(\theta_{y}- \hat\theta_{y})  $$

where $ \hat \theta _{x} $ and $ \hat \theta _{y} $ express the estimation of the angular variation of the joystick in the left/right and backward/forward directions respectively. *K*_*p*_ and *K*_*i*_ are the proportional-integral gains, $ \omega _{b_{x}} $ and $ \omega _{b_{y}} $ the gyrometer errors in the left/right and backward/forward directions respectively.

Complementary filters or Kalman filters are state-of-the-art in inertial-based system data fusion and attitude estimation [15–17]. The main idea is to rely on the accelerometer for a precise estimation in the static case and on gyrometer for the dynamic case [18]. Our complementary filter works as an association of a low-pass filter that captures long-term changes from the accelerometer and a high-pass filter that let short-term fluctuations from the gyrometer. The result is a better estimation of the inclination angle for each joystick axis.

### Joystick assessment metrics

In order to assess the driving performances of users, we borrowed seven indicators from [6] to characterize the actions on the joystick. Those are the excursion, the number of joystick actions, the total time, the direction, the mean joystick direction, the standard deviation of the direction and the mean velocity of EPW.

The joystick excursion is estimated as:

5 $$ D_{inv}=\sqrt{X^{2}+Y^{2}}  $$

for direct joystick outputs case and

6 $$ D_{ninv}=\sqrt{{\hat\theta_{x}^{2}}+{\hat\theta_{y}^{2}}}  $$

for the SenseJoy one. The joystick direction is computed as:

7 $$ \theta_{inv}=\arctan \frac{Y}{X}  $$

in the direct joystick outputs case and

8 $$ \theta_{ninv}=\arctan \frac{{\hat\theta_{y}}}{{\hat\theta_{x}}}  $$

otherwise. The number of joystick actions is defined as the number of times where the joystick excursion exceeded the threshold of 10% maximum amplitude from the joystick’s center position during a trial. The total time required to execute each trial is determined as the time from the first joystick action to the last. The direction is computed as a vector with a unique origin positioned on the joystick’s center. Each vector determines the direction for a sample. The average joystick direction is the average vector. The standard deviation of the direction vectors is also estimated. The velocity of the EPW in backward/forward direction is calculated by integrating the linear acceleration on the Y-axis of the accelerometer 2.

### Protocols

Two measurement campaigns have been made with different drivers and wheelchair using the same experimental protocol. A first data logging collection is conducted in a lab environment on a unique EPW equipped with SenseJoy and with a direct monitoring of the joystick outputs. This first measurement campaign is designed to make a thorough comparison with ground truth recording, with a direct joystick outputs setting, to validate the signal quality and the accuracy of the measurements. A second measurement campaign is conducted in a rehabilitation center on the personal EPW of individuals equipped with SenseJoy only. This second set of measurements is designed to demonstrate and to validate that our system is pluggable and compatible with any EPW driven by a joystick interface.

**Fig. 4:**
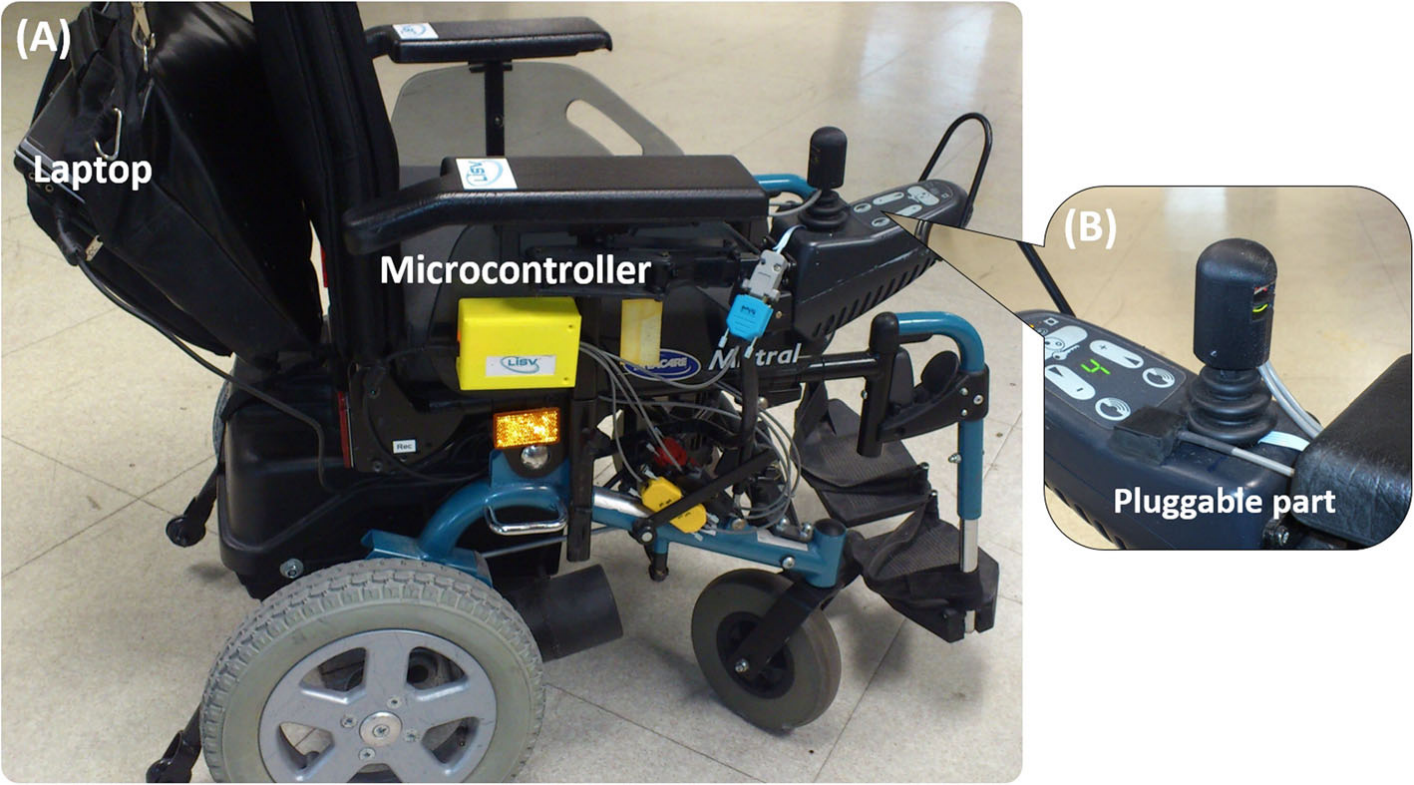
Lab EPW with SenseJoy. **a** Invacare’s EPW (Mistral model) equipped with the SenseJoy system. **b** Enlargement of the joystick part showing the pluggable part

The EPW used in the first measurement campaign comes from Invacare, it is a "Mistral" model. It has a rear-wheel drive system and a bidirectional joystick control interface placed on the right side, as shown on Fig. 4. The EPW is equipped with SenseJoy and the joystick was disassembled, as in [6], in order to monitor the two joystick analog outputs (X,Y). This allows a precise comparison with the ground truth recording (direct joystick output setting) to validate the signal quality and the accuracy of the measurements. For the second measurement campaign conducted in a rehabilitation center, our system is just plugged on the personal EPW of subjects, without requiring a joystick disassembling.

**Fig. 5:**
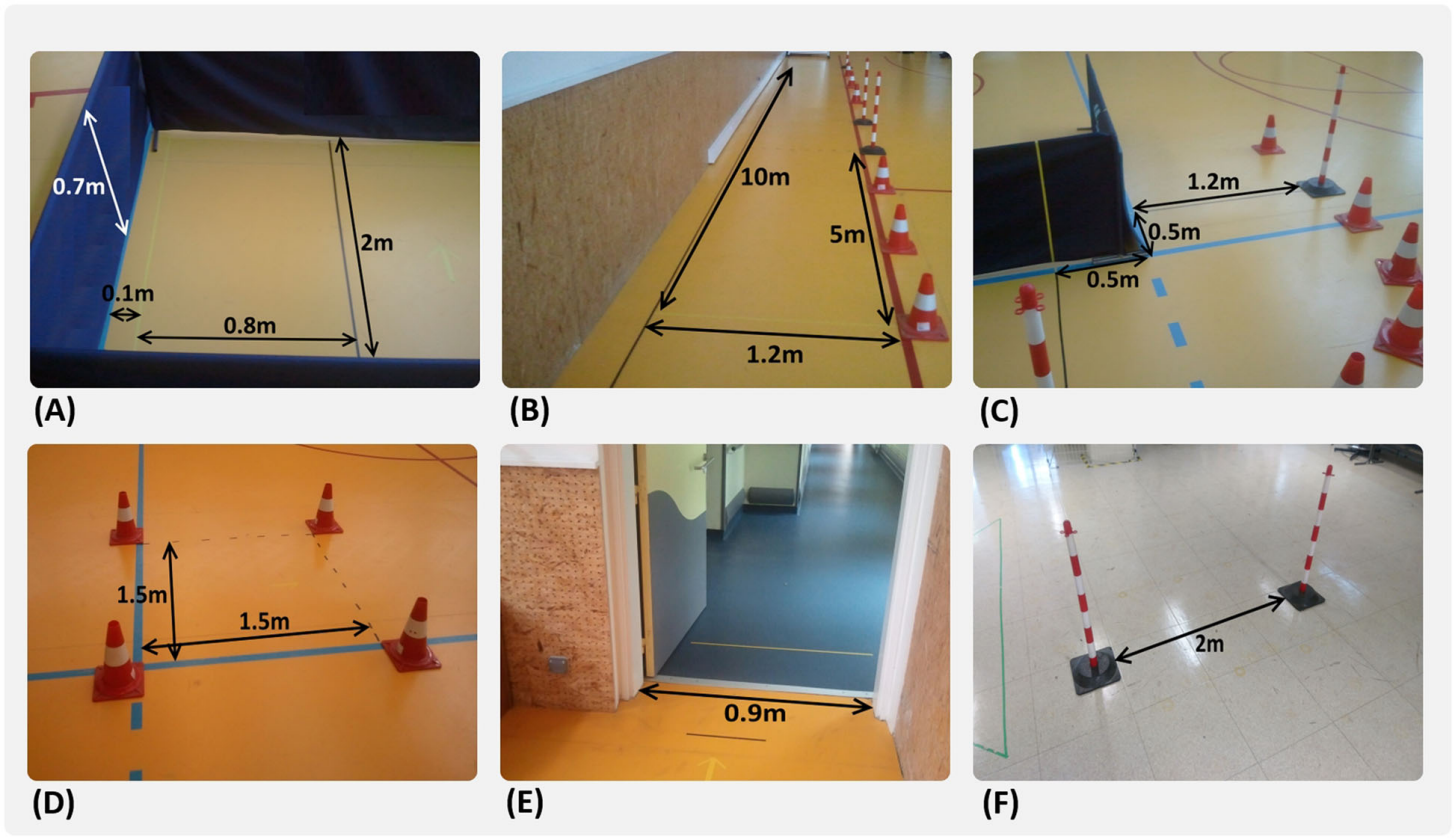
Six workshops designed to define the 13 exercises. **a** Sideways maneuvers (left and right), **b** straight trajectories (rolls backward on 5 m and rolls forward on 10 m), **c** turns 90 ∘ while moving (forward and backward; right and left), **d** turns 180 ∘ in place (right and left), **e** gets through hinged door in both directions, **f** slaloms between two studs

We apply the same experimental protocol in each measurement campaign, based on the WST [9] because it is a gold standard in research and clinical studies. Our experimental protocol is designed by identifying and selecting the tasks from WST protocol that are the most significant for the daily activities of an EPW driver: straight trajectories, turns, U-turns, maneuvers, etc. Only one exercise, the slaloms between two studs, does not belong to the WST protocol. It is added to observe the combined effect of alternating straight trajectories and curves, that generate difficult perturbations for the SenseJoy sensors. Thus, each participant has to complete thirteen exercises detailed in Table 1, distributed among six workshops as shown on Fig. 5. A participant needs on average 45 min to complete all exercises. Instructions are given orally to participants before each trial.

**Table 1:** List of the thirteen exercises of the proposed protocol

Order	Exercises
1	rolls forward for 10 m
2	rolls backward for 5 m
3	turns left 90 ∘ in forward direction
4	turns left 90 ∘ in backward direction
5	turns right 90 ∘ in forward direction
6	turns right 90 ∘ in backward direction
7	maneuvers sideways left
8	maneuvers sideways right
9	turns left 180 ∘ in place
10	turns right 180 ∘ in place
11	gets in through a hinged door
12	gets out through a hinged door
13	slaloms between two studs

### Cohort

The analyses are carried out with two populations. The objective of the first measurement campaign is to compare the performance of the direct joystick outputs system and the SenseJoy solution. It is conducted in the lab, with 8 individuals (labeled D1 to D8) that are assessed on the EPW shown on Fig. 4. The mean age in years of the group is 31.3±8.0 years. The subjects gathered six valid persons with no experience in EPW driving and two drivers with disabilities: an experienced user who drives an EPW daily (D5) considered as an expert and a novice EPW user (D2) not requiring EPW or manual wheelchair. Pathology of driver D5 is cerebral palsy and it is left hemiplegia for D2. Expert level is considered as more one year of EPW driving experience at the time of testing.

The objective for the second measurement campaign is to test the robustness and precision of our SenseJoy system, in an ecological context associating disabled EPW users with different levels of practice. Five individuals (labeled D9 to D13), mean age in years of the group is 41.4±10.5 years, have been recruited in a rehabilitation center. They are assessed on their own EPW equipped with SenseJoy, therefore there is no ground truth value for these recordings.

Table 2 shows a demographic summary of subjects who participated in the measurement campaigns. Both campaigns were conducted in accordance with the relevant guidelines for ethical research according to the Declaration of Helsinki. All the participants signed an informed consent form at the beginning of the experiment. The tests in a rehabilitation center where conducted under the medical supervision and validation of the center medical team.

**Table 2:** Demographic summary of subjects, group 1 gathers 8 drivers (D1 to D8) assessed in a lab environment and group 2 gathers 5 drivers (D9 to D13) recruited in a rehabilitation center and are assessed on their own EPW. The drivers are either novices, or experts or novices with Cognitive Impairment (CI)

Groups	Subjects	Gender	Age	EPW model	Diagnosis	Level
	D1	F	29	Invacare Mistral	-	Novice
	D2	M	43		Left hemiplegia	Novice
	D3	M	29		-	Novice
Group 1	D4	F	23		-	Novice
(analyses 1	D5	F	26		Cerebral palsy	Expert
& 2)	D6	M	48		-	Novice
	D7	M	33		-	Novice
	D8	M	31		-	Novice
	D9	F	53	Invacare Dragon	Right hemiplegia	Novice with CI
Group 2	D10	F	44	Invacare Mistral	Stroke	Novice with CI
(analysis 2)	D11	M	46	Icare Partner	Tetraplegic with cogn. dis.	Expert
	D12	M	39	Invacare Dragon	Tetraplegic	Expert
	D13	M	25	Levo C3	Tetraplegic	Expert

## Results

### Calibration and measurement characteristics

**Fig. 6:**
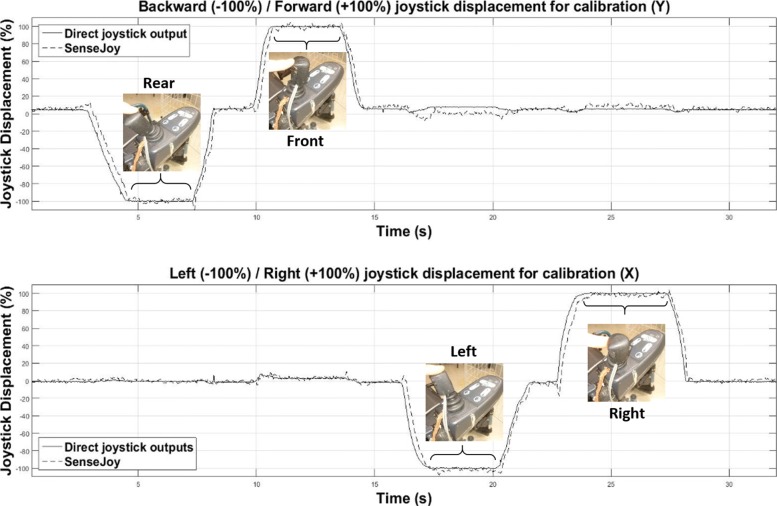
The SenseJoy system calibration steps for the two joystick axis. The calibration requires to place the joystick in the maximum rear position and then in maximum front position follow by the maximum left position and the maximum right position. Data are recorded during this process, the mean values of each maximum and minimum phases defining the limits

Before using SenseJoy, a calibration step is necessary to determine the maximum amplitudes of the bidirectional joystick of each EPW. The calibration step is conducted to ensure the correct parameterization of the offline analysis. The joystick is positioned in the maximum rear position and then in maximum front position followed by the maximum left position and the maximum right position, as visible on Fig. 6. Data are recorded during this process and the mean values of each maximum and minimum phases act as bounds to limit the joystick amplitude. This step allows to normalize joystick displacement values. Note that the rest position of the joystick for the backward/forward axis (Y) is not exactly zero on Fig. 6: for this axis the joystick is slightly inclined forwards in the rest position.

We can deduce two metrological parameters, resolution and response time. The resolution is defined by the standard deviation of the measurement noise. For each axis X and Y, the system is assumed to be recorded in static position. The recorded data is thus assumed to be characteristic of the static position with an additional white noise, due to the measurement chain. After verifying that the noise follows a Gaussian distribution, the standard deviations are respectively estimated to 1.31% and 1.23% for the X- and Y-axis. The response time is measured to be the time of the sensor response to reach 95% of the step. It has been measured averaged over multiple samples and is estimated to 95 ms.

### First analysis: comparison with direct joystick outputs

**Fig. 7:**
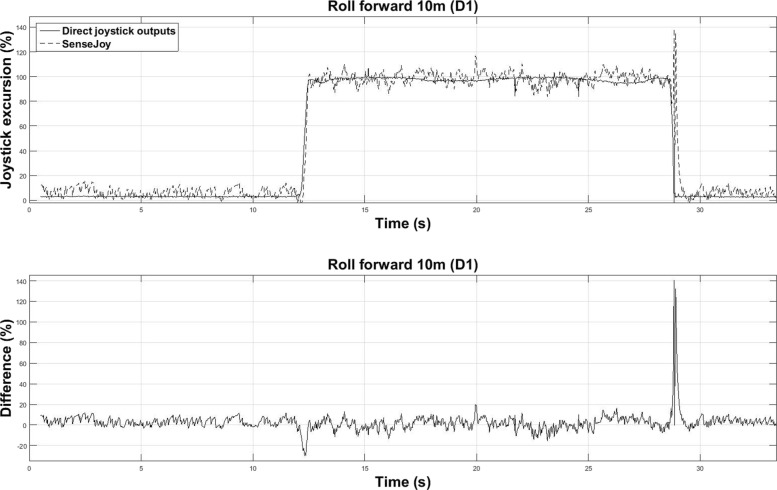
Example recording of a joystick excursion during a roll forward trial (driver D1). (Top) direct joystick outputs and SenseJoy system recordings. (Bottom) error as the difference between them. This difference follows a Gaussian distribution (SD: 8.8%)

The SenseJoy equipment includes Micro Electro Mechanical System (MEMS) sensors that measure linear accelerations and angular velocities. In consequence the dynamic of the wheelchair has a noticeable impact on the joystick angular estimations. Figure 7 illustrates a typical joystick excursion during a roll forward exercise over 10 m done by driver D1. This exercise is a rectilinear movement in forward direction, aligned with the Y-axis of the accelerometer sensors. In this case, removing the acceleration of the wheelchair from the joystick as in Eq. (1) allows to estimate the joystick angle with a good accuracy. Peaks may appear when the driver releases suddenly the joystick, as it can be observed near 30 s on Fig. 7. These peaks have limited impact on the offline analysis, a carefully tuned filter could filter out the peaks but the cut frequency must be correctly chosen to avoid removing useful information.

**Fig. 8:**
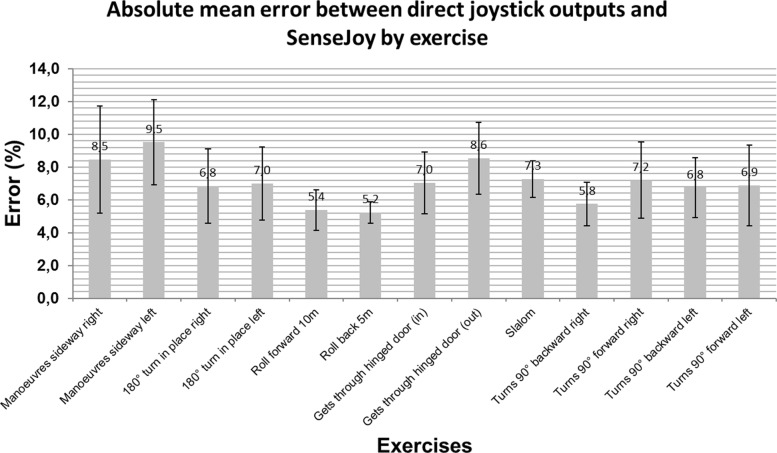
Comparison of mean absolute errors per exercise. Those are average values for the 8 drivers of group 1

Figure 8 displays the absolute mean error between direct joystick outputs and SenseJoy excursion for each exercise and all drivers of group 1. The rolls forward over 10 m and the rolls backward over 5 m exercises yield the lowest absolute mean errors (5.4% and 5.2% respectively) because movements of the joystick remain simple and are in accordance with our mechanical model as explained before.

**Fig. 9:**
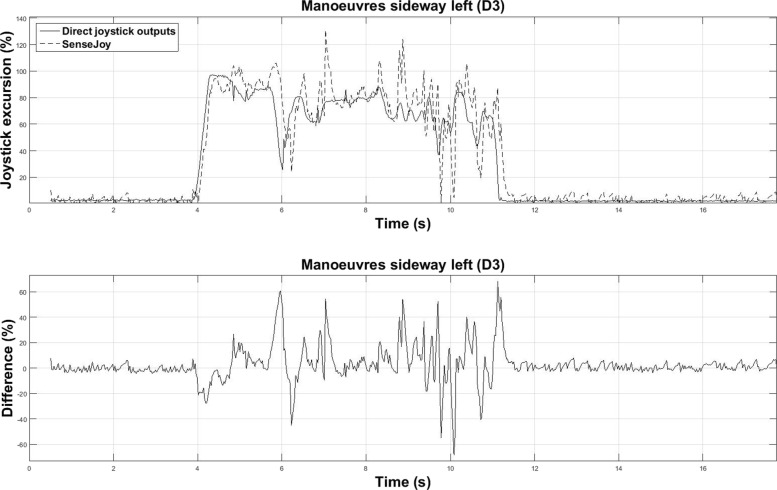
Example recording of the joystick excursion during a complex maneuver (a left sideway maneuver of driver D3). (Top) Direct and SenseJoy system recordings, (Bottom) error as the difference between them. This difference follows a Gaussian distribution (SD: 13.4%)

For more complex exercises with more actions on the joystick several sources of error could impede the joystick angle estimations, for example: prediction error, overshoot, noise, peaks or delay time, all of which are visible on Fig. 9. One can see a slight delay between both sensors due to filtering. This delay is not an issue because the data are analyzed off-line.

Prediction errors made by the SenseJoy system is imputable to the simple yet limited model of the system, which relies on a direct subtraction of the two accelerometer components. A direct subtraction is not accurate for movements combining rotations and translations and the wheelchair acceleration is only partially removed from joystick acceleration. A more advanced mechanical approach could improve the results but this version provide suitable results as they are close to the baseline directly measured on the joystick.

**Fig. 10:**
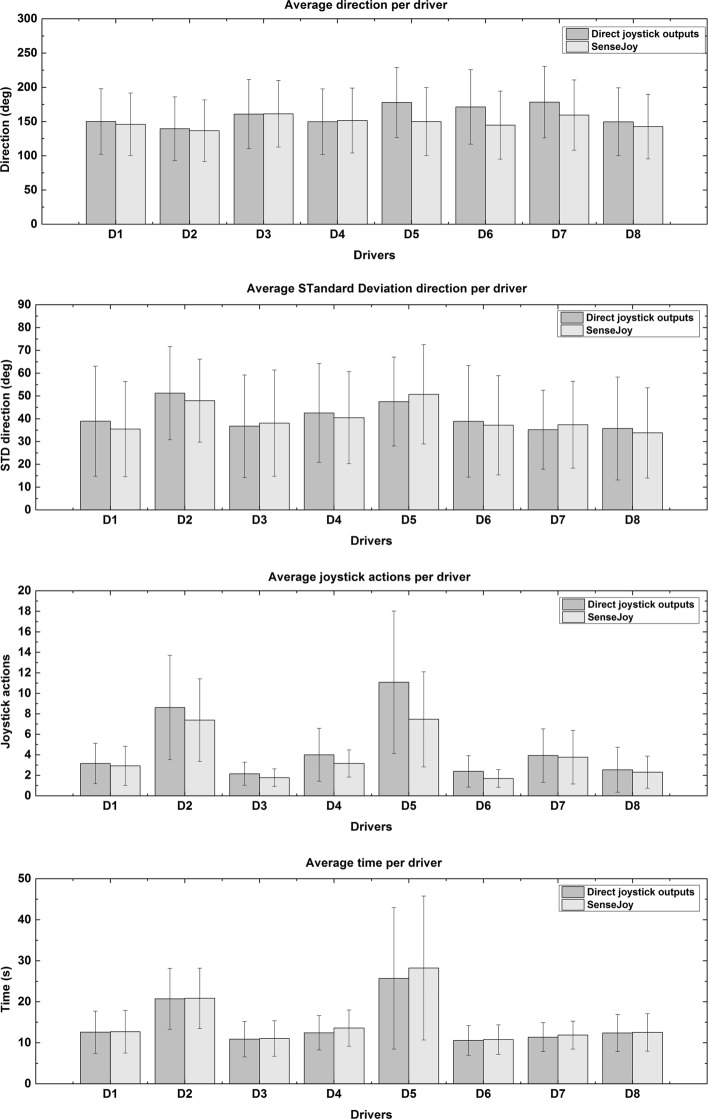
Comparison between direct joystick outputs and SenseJoy system per driver. Average of indicators per group 1 drivers for the thirteen exercises. D2 and D5 show deviations from the rest of the driver for average joystick action and average time per driver. The results for all indicators and for each driver are statistically equivalent, according to TOST procedure (*p*<0.05).

In order to properly evaluate SenseJoy against a baseline obtained from direct measurements, we averaged each indicator by users on Fig. 10, which are the direction, the standard deviation of direction, the number of actions and the total time. The direct measures and SenseJoy means are similar for all indicators and all drivers. The standard deviations are high as the results are aggregating the 13 exercises and there is a high variability between the exercises. To validate that the two systems generate comparable results, we must assert that the measurements between the baseline and the SenseJoy systems are not statistically different. Common statistical tests can ensure that a difference is significant but cannot help to conclude if two sets of measurements belong to the same distribution. Thus, we rely on the Two-One-Sided t-Tests (TOST) methodology [19, 20], a robust method which is recommended by the American Food and Drug Administration (FDA) to establish bioequivalence. This allows to evaluate the equivalence between a target population of *η*_*T*_ elements characterized by its mean and standard deviation (*μ*_*T*_,*σ*_*T*_) and a reference population of *η*_*R*_ elements characterized by (*μ*_*R*_,*σ*_*R*_). By defining a region bounded by a lower *Δ*_*L*_ and an upper *Δ*_*U*_ bounds, we could test a composite null hypothesis that combines *H*0_1_={*μ*_*T*_−*μ*_*R*_≤*Δ*_*L*_} and *H*0_2_={*μ*_*T*_−*μ*_*R*_≥*Δ*_*H*_} hypotheses. If we could reject both *H*_1_ and *H*_2_, we could conclude that *Δ*_*L*_<*μ*_*T*_−*μ*_*R*_<*Δ*_*H*_ and that the considered measurements are close enough to be considered as equivalent [21]. Following the work of [22] to set *Δ*_*H*_=−*Δ*_*L*_ with objective values, they proposed to use $\Delta _{H}=\lambda (\frac {\frac {1}{2}\sigma _{T}+\frac {1}{2}\sigma _{R}}{\sqrt {\eta _{T}+\eta _{R}}})$ with *λ*=4.581. We considered tighter bounds with *λ*=2 to ensure a stricter equivalence in our study.

The following results are obtained: for average joystick actions *Δ*_*H*_=0.2 and *p*≈1×10^−5^, for an average time *Δ*_*H*_=1.5 and *p*≈3×10^−4^, for average direction *Δ*_*H*_=4.7 and *p*≈2×10^−3^ and for average standard deviation of direction *Δ*_*H*_=5 and *p*≈1×10^−5^. We can conclude that for all indicators SenseJoy yields results that are equivalent to the direct reference.

### Analysis 2: driver profile clustering

**Fig. 11:**
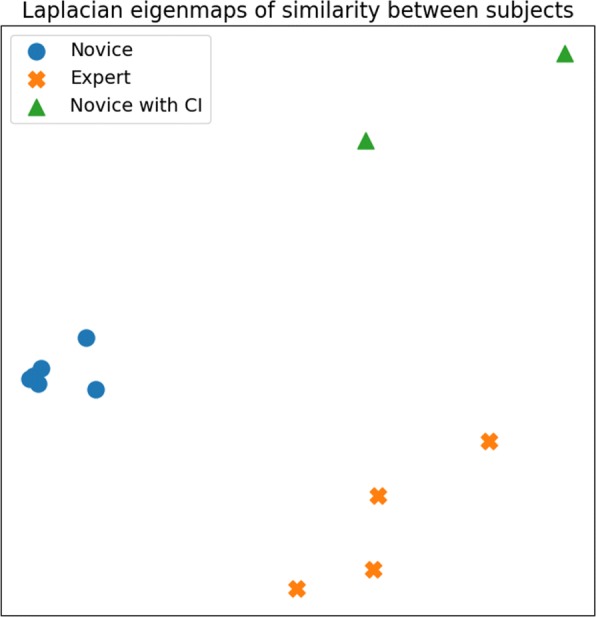
visualization of the similarity between drivers. Laplacian eigenmaps of similarity between drivers of the groups 1 and 2, computed over all exercises. Three groups of driving behaviors are visible: a group of novices (blue), a group of novices with cognitive impairment (CI) that affected the driving abilities (green) and a group of experts in EPW driving (orange)

The driver behavior could be characterized by the indicators derived from the joystick interactions [12]. We propose a novel approach that relies on these indicators to design an automatic clustering procedure. Clustering methods partition the parameter space to identify groups with similar behaviors. Nonetheless, clustering methods assume that the input space is consistent across all subjects, that is not the case here as the indicators (joystick action, time,...) are not homogeneous between exercises. A proper clustering method for the driver characterization should adopt an ensemble approach to take into account the exercise variability. For this second experiment, we propose a new clustering procedure based on the indicators estimated during the 13 workshops. A *k*-means clustering with *k*=4 classes and 300 iterations is conducted on each exercise using the indicator values as input. These values are chosen for their robustness, i.e. adding more iterations or clusters do not qualitatively change the results. A similarity matrix *S* is built by counting in the number of exercises for which two drivers are in the same cluster and by dividing the results by the total number of exercises. If we denote by *η*_*E*_ the number of exercises and by $K_{u}^{e}$ the cluster returned by the *k*-means for the user *u* and the exercise *e*, the similarity matrix *S*_*ij*_ entries are defined by:

9 $$ S_{ij}=\frac {1} {\eta_{E}}{\sum_{}}_{e}\delta_{K_{i}^{e}K_{j}^{e}} \;\;if\;\;i\neq j \,,  $$

where *δ*_*xy*_ is the Kronecker delta function, that is equal to 1 if *x*=*y* and 0 otherwise. This matrix *S* holds all the information regarding the similarity between two drivers across all considered exercises. For this second experiment, we have estimated *S* for the *n*_*E*_=13 exercises and for all the drivers of the groups 1 and 2. As this matrix account for high dimension data, a dimensionality reduction method could be applied to visualize the results. We choose the Laplacian Eigenmaps [23] to process the similarity matrix *S* as it is best suited to work with similarity matrices. This clustering method shows on Fig. 11 three groups of drivers that are in accordance with the profiles of each group. A cluster of novices (in blue on Fig. 11), a cluster of novices with cognitive impairment that affect the driving (in green) and a cluster of experts in EPW driving (in orange).

## Discussion

In this study, SenseJoy is designed and 3D-printed as a stick with an external diameter of 30 mm. While standard diameters are usually smaller, circa 20 mm, we could not meet this condition due to the size of the electronic card for the inertial sensors. As we rely on retail available hardware and components, it was not possible to reduce significantly this electronic part. Future development of this system will rely on miniaturized components to be embedded in standard diameters.

The first experiment shows on Fig. 7 and 9 that the signals are very similar even if the SenseJoy signals are not as clean as the real joystick signal. In order to use the accelerometers as inclinometers, we made the hypothesis the wheelchair acceleration is small compared to $\vec {g}$, the gravitational force on Earth. This is a strong requirement to ensure the quality of the estimation made by our system. Many EPW exist on the market with various characteristics (velocities, maximum weight of users, dimensions, etc.) and their acceleration are not an easily accessible data from the constructors. Velocity is commonly indicated as a main parameter of powered wheelchairs. For a standard EPW, the velocity is usually limited at 6 km/h or 10 km/h, while faster ones can reach 23 km/h. It is difficult to determine the acceleration of EPW from velocities. To provide a meaningful example, a sports car that is able to reach 100 km/h in 4 s is subject to an acceleration equivalent to 6,94 m/s^2^ and this value is still smaller than $\vec {g}$. We could thus reasonably consider that our hypothesis holds for the commercially available EPW and that we could use accelerometers as inclinometers.

The estimation of the joystick angle is obtained with a crude computation to remove the wheelchair acceleration. While this approach is correct in the case of the rectilinear movement, more complex driving situations combining rotation and rectilinear movement are more error prone for the system. To ensure that our system is robust, we add a specific exercise to generate these situations, that is the slaloms between two studs. The average results per exercises on Fig. 8 and per driver for each indicator on Fig. 10 shows that the system provides a correct estimation when compared to the direct output of the joystick. The statistical validation using an equivalence test also indicates that our pluggable system is able to correctly estimate the joystick movement, even in the case of complex driving situations.

Even if SenseJoy is equivalent from a statistical point of view to the direct recording of a joystick, some discrepancy are visible under close examination. The high variability of the signal requires to apply filters to remove noise sources and perturbations. We propose to rely on specific filtering method, called complementary filter, that is known to work well on systems like SenseJoy. Even if the complementary filter is well adapted for the joystick measurements, all filters remove some informative data. This could be seen on the joystick actions shown Fig. 9. With the direct measure from the joystick, the driver D5 has an average of 11.1 actions per workshop, while only 7.5 actions are detected by SenseJoy. The driver D5 is a subject with more than a year of EPW driving experience that we could record with both direct measures and SenseJoy. While these differences are visible, they are not statistically significant and D5 driving style is recognized as “expert” by the unsupervised clustering method. This limitation of the system could be harnessed with a continuous evaluation of the driver, that is repeating the driving tasks on several days to obtain average measurements.

For the second experiment, the objective is to validate that the precision of SenseJoy allows to characterize the user behavior and is able to detect the expertise in EPW driving. One can note that the experiment is conducted on the two groups of drivers that have been recorded in two different locations. On Fig. 1, it is interesting to note that the expert driver recorded in the group 1 belongs to the same cluster as the experts recorded with the group 2. This demonstrates that this clustering method is robust to the location change and could be used for larger cohort analysis. The two novices with cognitive impairment in group 2 form a specific cluster due to their specific driving style. Whereas all novices of group 1 are together, we can observe that there is two outliers, one of them is D2 who had a slower driving style due his pathology, the other one is a very cautious driver that was very careful in her mobility.

## Conclusion

In this study we propose an easy-to-plug equipment to assess the joystick controls during EPW driving tasks. This equipment can be plugged on any EPW equipped with a joystick control interface. We have demonstrated it is possible to collect the data and to estimate the driving indicators from the literature. These indicators help caregivers in rehabilitation centers to objectively quantify the driving behavior based on the direct measure of joystick actions. We have demonstrated that our system is robust and accurate with thorough and ecological tests. It is comparable to the direct output of the joystick, both quantitatively, as it is validated by a statistical equivalence test, and qualitatively, as we could extract the expert/novice driving profile with an automatic clustering method. This is an important advance as previous studies relying on the analysis of the joystick behavior required a dismantling procedure to directly record the joystick signal, a procedure that could not be conducted in rehabilitation centers. Our system allows to record any EPW without any destructive procedure and provides the opportunity to test a large cohort of EPW drivers in an ecological context.

There are several interests for pluggable system like SenseJoy. It allows to evaluate the capability and usage-related performances of a driver using the subject equipment without altering it. These ecological measurements allow to identify driver profiles, as described in [12]. It is thus a good basis for assessing supervised training and continuous learning. As an identification tool for driving profile, it is complementary to clinical indicators. The main advantage is that it is an embedded system, that could be applied to wider use case for the monitoring of daily living activities.

It is limited in its present design to specific situations, that is a joystick-controlled EPW, as other types of controller like touch interface, sip-and-puff or forehead/chin joystick are not supported. Also, SenseJoy may not be suited for subjects with motion-impairement that exhibit spastic movements. This could be the subject of following evaluation to investigate those points.

The actual version could be improved by a mechanical approach avoiding the impact of the EPW movement on the joystick actions measurements. We also plan to develop other pluggable tools to measure other driving-related data, thus completing the analysis of EPW driver’s profile.

## Data Availability

The dataset used and/or analyzed during the current study are available from the corresponding author upon reasonable request.

## Abbreviations

EPW: Electric Powered Wheelchairs
FDA: Food Drug Administration
MEMS: Micro Electro Mechanical System
TOST: Two-One-Sided t-Tests
WST: Wheelchair Skill Test
